# Liver and Kidney Tissues Under Opioid Exposure: Rewriting and Old Story Through Proteomics and MALDI–MSI

**DOI:** 10.1021/acs.jproteome.5c00764

**Published:** 2026-01-24

**Authors:** Malgorzata Hopcias, Paulina Kret, Jolanta H. Kotlinska, Pawel Link-Lenczowski, Anna Bodzon-Kulakowska, Piotr Suder

**Affiliations:** † Department of Analytical Chemistry and Biochemistry, 49811 AGH University of Krakow, Mickiewicza 30 Avenue, 30-059 Krakow, Poland; ‡ Department of Pharmacology and Pharmacodynamics, Medical University of Lublin, Chodzki 4A St., 20-093 Lublin, Poland; § Department of Medical Physiology, Faculty of Health Sciences, Jagiellonian University Medical College, 31-126 Krakow, Poland; ∥ Center for the Development of Therapies for Civilization and Age-Related Diseases, Jagiellonian University Medical College, 31-066 Krakow, Poland

**Keywords:** liver, kidney, morphine exposure, MALDI imaging, DDA/DIA MS analysis

## Abstract

A proteomic analysis of rat liver and kidney was performed following exposure to morphine (10 mg/kg, 10 days) versus untreated controls. Our data revealed alterations in numerous protein expression. Differences were observed in pro- and antiapoptotic signaling, oxidative stress response, transport mechanisms, nucleic acid metabolism, and regulators of alternative splicing, among others. Liver tissue exhibited primarily adaptive changes, including upregulation of metabolic enzymes and subtle markers of oxidative stress. In contrast, the kidneys displayed a broader spectrum of proteomic alterations, affecting a wider array of functional pathways, suggesting that this organ may be more susceptible to morphine-induced toxicity or that of its metabolites. We employed also MALDI–MSI to examine lipidomic alterations: while no significant changes were detected in liver tissue, important lipidomic alterations were observed in the kidneys, further supporting their increased vulnerability to morphine exposure. Our results should be regarded as preliminary; however, they highlight a promising direction for identifying early stage protein markers of toxicity using proteomic approaches. Monitoring such markers could prove valuable in multidrug therapies, particularly for patients with a history of hepatic or renal impairment, contributing to the development of safer therapeutic strategies. Data are available via ProteomeXchange under the identifiers: PXD067999 (DDA) and PXD068084 (DIA).

## Graphical abstract

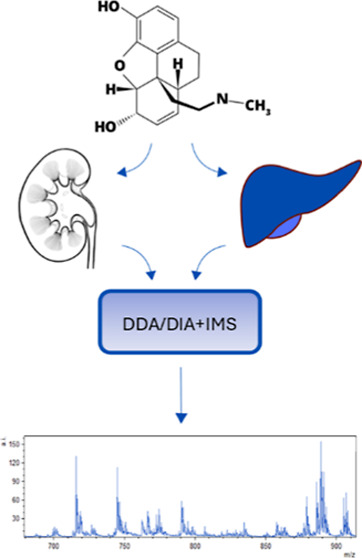

## Introduction

The liver and kidneys are the primary organs responsible for processing and eliminating substances and their metabolites from the body. During pharmacological intervention, the liver rapidly converts drugs via phase I and phase II metabolic reactions, which usually reduce the compound’s toxicity. The kidneys, acting as the primary plasma filtration units, remove drug metabolites produced by the liver, as well as unchanged drug forms, and excrete them into the urine through the renal glomeruli. This evolutionarily optimized mechanism is highly effective for many compounds present in the human environment. The metabolic reactions occurring in the liver during xenobiotic processing are well characterized, and detailed descriptions are widely available in the literature.^1^ Similarly, the principles governing renal function are comprehensively described in numerous publications.^2^

In the pharmaceutical industry, any substance being tested as a potential drug must undergo rigorous ADME (Absorption, Distribution, Metabolism, and Excretion) testing during the preclinical phase, according to standardized procedures such as GLP, GMP, and OECD guidelines. Based on current pharmaceutical standards, medications approved for therapy are generally considered safe when administered according to established protocols. However, drug interactions in polypharmacy scenarios often complicate the prediction of organism/drug responses. Some interactions between major drug groups cannot be anticipated during preclinical testing and are only discovered in phase I or II clinical trials. Considering that modern pharmacotherapy involves at least 2000 active substances (based on data from the World Health Organization (WHO), European Medical Agency (EMA), Food and Drug Administration (FDA), DrugBank, and ChEMBL), comprehensive cross-testing for undesirable tissue interactions remains difficult, if not impossible.

Our team has been studying the role, side effects, and atypical actions of opioids in the central nervous system, using morphine as a reference compound. Morphine is the gold standard opioid in pain management but is also a commonly abused substance. Its pharmacology, side effects, and metabolism are well documented in numerous scientific and pharmacological publications. After peripheral administration, morphine slowly crosses the blood–brain barrier and interacts with μ-opioid receptors (μ1 and μ2, also known as MOR1 and MOR2), leading to the inhibition of pain perception. Like other opioids, including heroin, fentanyl, and oxycodone, morphine is also associated with a high potential for addiction and other serious health issues. In addition to its analgesic effect, morphine like other opioids, has several side effects, resulting from its nonspecific interactions with other cells in the body. In addition morphine metabolites have a range of actions on cells or tissues, even though they lack pain-relieving activity.

Morphine is minimally metabolized in the central nervous system; instead, it is processed primarily in the liver. In hepatic tissue, morphine is converted into three major metabolites: morphine-3-glucuronide (M3G), morphine-6-glucuronide (M6G), and normorphine (the *N*-demethylated form). Other minor metabolites, such as M3,6-diG and morphine-3-ether sulfate, are produced in small amounts. A minor fraction of morphine is excreted in the unchanged form. The enzymes primarily responsible for morphine metabolism in hepatocytes include UGT2B7, which catalyzes glucuronidation to M3G and M6G, and cytochrome P450 isoforms CYP3A4 and CYP2C8, which catalyze *N*-demethylation to normorphine. Notably, according to some reports, morphine may also be metabolized in the kidneys, with renal metabolism accounting for up to 38% of the administered dose.^3^

It is widely recognized that morphine and other opioids are generally well tolerated by the liver^4^ and kidneys^1^ during standard pharmacological treatment. However, the effects of chronic opioid exposure to high doses, such as in opioid abusers, remain underinvestigated. In these cases, observed liver and kidney damage is often attributed to confounding factors such as viral infections, contaminated substances, or hazardous lifestyle conditions. In addition, multidrug therapy, involving morphine, opioids or even the general class of analgesics combined with other drugs, can significantly, but in some cases in an unknown manner, influence the homeostasis of the liver and kidneys, slowly leading to the degradation of both organs. Even for separate opioid intake, some studies have reported links between long-term opioid use and specific pathologies, including nonalcoholic fatty liver disease (NAFLD), renal fibrosis, organ cirrhosis, and other, less frequent syndromes.^5–7^ Based on the available literature, prolonged exposure of both organs to morphine, fentanyl, and other opioids, even if various opioids have slightly different metabolic pathways, results in oxidative stress-related damage.^8–10^ During our previous investigations focused on the effects of morphine on the central nervous system and spinal cord,^11,12^ we noted a surprising lack of published data on the impact of morphine on liver and kidney tissues, particularly in proteomic investigations. Proteomics is a valuable tool for investigating the early stages of pathophysiological process before their detectable manifestation in the organism, enabling a deep understanding of the molecular fundamentals underlying later organ or tissue damage. The role of proteomics cannot be underestimated in modern investigations in physiology, toxicology, and other branches of the life sciences; hence, this study aims to enhance understanding of opioid interactions in the liver and kidneys, using morphine as a gold standard. Our findings will contribute to understanding the molecular basis of organ reactivity, even if our investigations are considered preliminary. They can serve as a foundation for selecting markers of early tissue damage in complex pharmacotherapies, including opioids. To comply with the 3R principle (replacement, reduction, refinement) in animal research and taking advantage of stored liver and kidney samples from laboratory rats, we examined the proteomic response of these organs to short-term morphine exposure. Although the presented data are preliminary, they clearly indicate a more complex tissue reaction to morphine administration than is typically described. To potentially gain insight into changes occurring in the investigated tissues, we assessed whether there are more global changes in lipid composition in both organs following morphine exposure, using MALDI–MSI to rapidly analyze lipid profiles from tissue sections.^13^ An additional advantage is the ability to indicate the spatial distribution of certain substances. In the case of the liver, it is less relevant since this tissue is very homogeneous. However, for the kidneys, the ability to identify distinct regions, such as the cortex and medulla, may be particularly important.

This study also complies with our previous investigations showing dynamic changes in the lipid composition of rats’ brains under the influence of selected drugs of abuse.^11^ In combination, our findings may explain specific processes occurring in the liver and kidneys that have so far remained obscure.

## Experimental Procedures

### Animals and Tissues Collection

All animal procedures were carried out in accordance with local and European regulations (Directive 86/609/EEC) and were approved by the Local Bioethics Committee for Animal Experiments at the Medical University Lublin (approval number 35/2020). A total of 20 male Wistar rats were housed in groups of three or four under a 12/12 h light/dark cycle, with free access to water and a standardized diet. The animals were maintained at the Center for Experimental Medicine, a unit of the Faculty of Pharmacology at the Medical University of Lublin, Poland. Morphine exposure was performed via intraperitoneal (i.p.) injections of morphine hydrochloride dissolved in saline at a dose of 10 mg/kg body weight. The control group received i.p. injections of saline alone, administered at the same time points and in the same volume as the morphine-treated group. The dosing regimen and morphine concentration were selected based on our previous experience and a thorough review of the literature. After 10 days, the animals were sacrificed by decapitation. Organs were immediately isolated, flash-frozen in liquid nitrogen, and stored at −80 °C until further analysis.

### Sample Preparation for Proteomics

Small fragments (weighing approximately 0.1–0.2 g) of liver were removed from the organs and transferred into 1.5 mL low-binding Eppendorf tubes. For the kidneys, similar tissue fragments were used, with special attention paid to obtaining a complete cross-section of the organ. Tissues were mechanically and ultrasonically homogenized in 0.75 mL of ammonia bicarbonate buffer (AMBIC, 50 mM, pH 8.0) containing 4% SDS and a protease inhibitor cocktail. All reagents and solvents were purchased from a local distributor of Merck-Millipore Corp. at the highest available purities. The homogenates were centrifuged 20,000 g for 10 min at room temperature (RT) and the supernatants were transferred into fresh tubes. The BCA assay^14^ was used to determine the protein concentration in each sample. Then, reduction (dithiotreitol, 5 mM, 20 min/80 °C) and alkylation (iodoacetamide, 5 mM 30 min, RT in the dark) were performed, followed by precipitation in ice-cold acetone overnight at −20 °C. A 7-fold higher volume of acetone was used compared to the sample volume. After supernatant removal, the pellets were dissolved in 150 μL of 50 mM AMBIC buffer, pH = 7.5. Trypsin (MS grade Gold, Promega, USA) was then added at a 1:50 (m/m) ratio, and all samples were incubated overnight at 37 °C with shaking. For DDA-based database preparation, equal amounts of each sample were mixed to obtain a total of 40 μL of solution containing 40 μg of peptides. After enzymatic cleavage, all samples were freeze-dried and dissolved in acetonitrile (ACN)/water/formic acid (HCOOH) (4:95.9:0.1, v/v/v) solution to obtain a final peptide concentration of 0.4 mg/mL.

### DDA-Based Database Preparation

The mixture containing all of the peptides from each sample was fractionated off-line into 17 fractions, each containing approximately 1 μg of peptides, according to the procedure described elsewhere.^15^ Briefly, the mixture was added to the reversed-phase (RP) column (Gemini, 100 × 1 mm ID, 3 μm 110Å, C18) and separated at a slightly alkaline pH. The solvents applied were as follows: *A* = 50 mM AMBIC + 2% ACN; *B* = 50 mM AMBIC + 80% ACN, both at pH 8.0. The gradient settings were as follows: time (*T*) = 0 min, 4% *B*; *T* = 39 min, 40% *B*; *T* = 40 min, 100% *B*; *T* = 49 min, 100% *B*; *T* = 50 min, 4% *B*; *T* = 51 min, 4% *B*. The flow rate was set to 40 μL/min with an injection volume of 17 μL. Each of the 17 fractions acquired. Each of the 17 fractions consisted of 3 × 1 min column eluate. The first fraction consisted of time frames: 0–1min + 17–18min + 33–34 min, the second: 1–2min + 18–19min + 34–35 min. Fraction acquisition continued until all of the 17 fractions were collected. Next, all fractions were freeze-dried and dissolved in 4% ACN/water solution acidified by HCOOH. Each fraction contained approximately 1 μg of peptides dissolved in 20 μL of the solvent (0.05 μg/μL).

The fractions underwent typical DDA in duplicate using a nano LC–MS/MS system consisting of an Ultimate 3000 capillary chromatograph connected online to an Exploris 240 mass spectrometer (both from Thermo Fisher Scientific, Bremen, Germany). The system was equipped with a Nanospray–Flex ion source. Precolumn/column setup was applied. The precolumn was PepMap Neo C18 300 μm/5 mm, and the analytical column was: Acclaim PepMap 75 μm/500 mm, both C18, 3 μm. The solvents: *A* = H_2_O + 0.1% HCOOH and *B* = ACN + 0.1% HCOOH. The gradient was: *T* = 0 min, 12% *B*; *T* = 4 min, 12% *B*; *T* = 83 min, 31% *B*; *T* = 84 min, 70% *B*; *T* = 85 min, 70% *B*; *T* = 86 min, 12% *B*; *T* = 90 min, 12% *B*. The flow rate was set to 300 nL/min. The mass spectrometer settings were as follows: (MS scans) resolution = 15,000, scan range of: 350–1300 *m*/*z*, time of acquisition: 8–90 min; RF lens 67%, ion transfer tube temp. 275 °C, positive ionization mode, voltage 2000 V; (MS/MS scans) resolution = 30,000, scan range of: 200–2000 *m*/*z*, HCD collision energy 36% and a threshold intensity of 5 × 10^4^.

### DIA Analysis

The DIA analysis was performed using the same equipment as the DDA. All freeze-dried samples were dissolved in a 4% ACN/water solution acidified with 0.1% HCOOH. The peptide concentrations in each sample were adjusted to achieve 0.1 μg/μL. For chromatographic separation, the same solvents *A* and *B* were used. The gradient was slightly shorter than applied for DDA: *T* = 0 min, 12% *B*; *T* = 4 min, 12% *B*; *T* = 74 min, 31% *B*; *T* = 75 min, 70% *B*; *T* = 76 min, 70% *B*; *T* = 77 min, 12% *B*; *T* = 80 min, 12% *B*. Mass spectrometer fragmentation settings were adapted from various literature sources. Briefly, the fragmentation range was divided into 40 windows spanning: 349.5–1150.5 *m*/*z*. A complete fragmentation cycle was no longer than 2.5 s, allowing for quantitation based on at least five points. The MS spectra ranged from 345 to 1650 *m*/*z*, with a resolution of 120,000. For MS/MS, the resolution was 30,000, the HCD collision energy was 28%, and the RF lens was 70%. All DIA analyzes were performed in duplicates.

An internal protein database using the DDA data was created with the aid of FragPipe,^16^ ver 22.0. The reference database used was: UP000002494 (*Rattus norvegicus*) containing 47,914 records (downloaded from uniprot.org). Quantitative analysis was performed in DIA-N′N software (ver. 1.9.2) using the previously created spectral library.^17^ Final data interpretation was processed by Perseus (ver. 2.1.0).^18^ Protein intensity values were normalized by median centering, log_2_-transformed and filtered to retain entries quantified in >70% of samples per group. Missing values were imputed using a downshifted normal distribution (width 0.3, downshift 1.8). Differential expression was assessed using a two-sample Student’s *t*-test with a permutation-based false discovery rate (FDR) control (250 permutations) implemented in Perseus (v.2.1.0). For the liver, differentially expressed proteins (DEPs) were defined as proteins with an FDR < 0.05 and S0 = 0.1; for the kidneys, a more stringent threshold of FDR < 0.01 and S0 = 0.1 was used. No arbitrary fold-change cutoff was applied, as significance thresholds naturally derive from the FDR/S0 framework. The STRING database (http://string-db.org) was used for the graphical presentation of protein–protein interaction networks involving only up- or down-regulated proteins against the database created from the proteins identified in the experimental procedure. The graphical interpretation of the volcano plots originally received in Perseus was provided by the VolcaNoseR web application.^19^

### Sample Preparation for MS Imaging

Before sectioning, the tissues were placed in a cryomicrotome chamber (Cryotome FSE, Thermo Fisher Scientific, Chesire, UK) to warm up. The chamber specimen temperatures were set to −15 °C. Slices were cut at 12 μm and immediately thaw-mounted on the on indium-tin-oxide (ITO) InteliSlide glasses (Bruker Daltonics, Bremen, Germany). Two identical sets of samples were taken: the first for the measurement in the positive-ion mode, the second for the negative-ion mode.

The matrices were applied to the tissue sections using the SunCollect sprayer system (SunChrom GmbH, Friedrichsdorf, Germany). The analysis parameters were chosen as described.^20^ In the positive ion mode, the DHB matrix (15 mg/mL, 50% MeOH, 0.2% TFA) was applied in 12 layers, with the spray nozzle at *Z* = 5 (high above the tissue). In negative-ion mode, a DAN matrix (2.5 mg/mL DAN, 50% ACN) was applied with 14 layers and a spray nozzle position at *Z* = 5 (high above the tissue). The flow rate for both matrix solutions varied across layers: 15 μL/min for the first layer, 20 μL/min for the second, 30 μL/min for the third, 50 μL/min for the fourth, and 60 μL/min for the remaining layers. Matrix solution spray deposition was performed linearly with a line distance of 2 mm and with a speed of 600 mm/min.

Matrix-coated sections were subjected to imaging experiments using a MALDI–TOF/TOF ultrafleXtreme MS system (Bruker-Daltonics, Bremen, Germany) with a Smartbeam II laser operating at 2 kHz. All MS parameters underwent initial, multistep optimization. Ions were accelerated at 25 kV with a pulsed ion extraction of 120 ns and ion suppression up to 100 Da. Spectra were recorded in positive and negative-ion modes with reflectron over a 100–3000 *m*/*z* range. The system was externally calibrated with Peptide Calibration Standard II (Bruker-Daltonics, Bremen, Germany) and known matrix ions.

For MALDI MSI, a raster width of 200 μm was used. In total, 400 shots were collected per spot, and the laser focus diameter was set to “3_medium.” FlexControl version 3.4 (Bruker-Daltonics, Bremen, Germany) was used for spectrum acquisition, and FlexImaging version 4.0 was used for data processing and molecular image creation. FlexAnalysis (version 3.4) was used for spectra analysis. All the data were normalized to total ion current (TIC).

### Partial Least Square (PLS) Analysis

Five control kidneys and five morphine kidneys were taken for analysis. For each tissue slice, three regions of interest (ROI) were taken from cortex, medulla, and the inner kidney. Each ROI summarizes nine laser spots. For the liver, six tissue slices for control and six tissue slices for morphine were taken.

Preprocessing and statistical analyzes were performed in MATLAB. Preprocessing was performed, including denoising using the MATLAB Mssgolay function (smooths the raw, noisy signal data using a least-squares digital Savitzky–Golay filter, SpanValue = 3), baseline correction using the MATLAB msbackadj function, (window size 250) and TIC normalization. Then, the spectra were assigned to the “control” and “morphine” group (−1 and 1). PLS regression was performed to determine the optimal number of latent variables (LVs) of the PLS model. This was achieved by identifying the lowest cross-validation (CV) error and the highest PLS variance for each analysis. In this way groups of the most important peaks discriminating between the two classes were indicated. Additionally, their presence was confirmed at a lower LV values.

## Results

DDA/DIA analysis identified 4281 proteins in liver tissue and 4653 proteins in kidney tissue corresponding to the top 70% of proteins (ranked by abundance and completeness of detection) and considered suitable for quantification in Perseus. Applying a threshold of FDR < 0.05 and S0 < 0.1, 205 proteins (approximately 5% of those selected for quantification) were found to be significantly deregulated in the liver. Among them, 139 proteins (68%) were upregulated, while 66 (32%) were downregulated. In the kidney, initial analysis with the same thresholds identified deregulation of 953 proteins (20%). To increase specificity, more stringent criteria were applied (FDR < 0.01, S0 < 0.1), resulting in 381 significantly deregulated proteins, of which 196 (52%) downregulated and 185 (48%) upregulated. Figure 1 shows volcano plots illustrating the distribution of significantly regulated proteins in the liver and kidney tissues.

**Figure 1:**
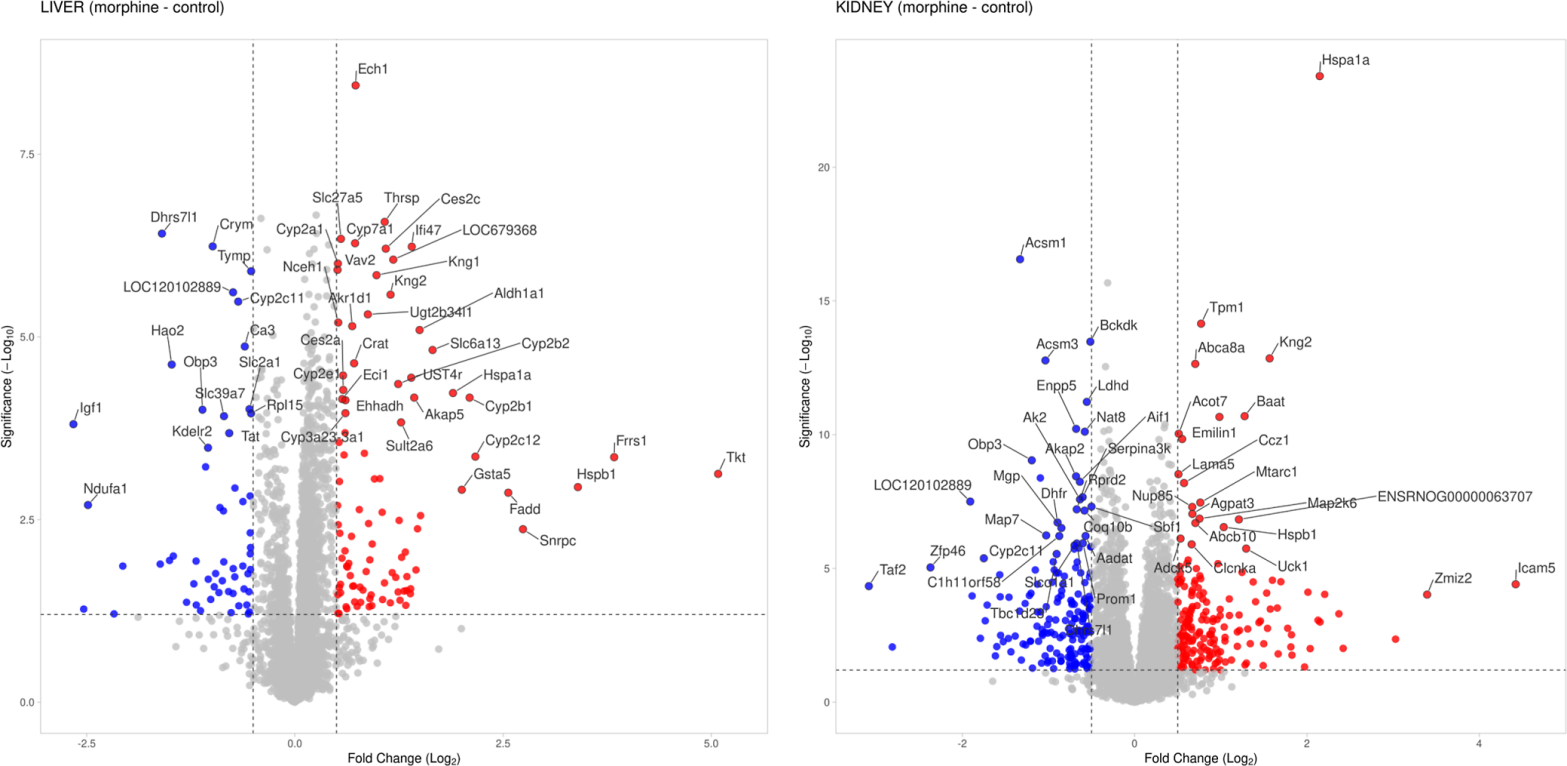
Volcano plots of proteins deregulated in morphine-exposed liver (left panel) and morphine-exposed kidney (right panel) compared to control tissues. Red dots: upregulated proteins, blue dots: downregulated proteins. The top 50 most significantly regulated proteins are shown on each panel.

### Proteins Deregulated in the Liver

A detailed list of all proteins deregulated at FDR < 0.05 and S0 < 0.1 is provided in the Supporting Information (S1: S1_table_1.xlsx). These proteins can be categorized into major functional groups: metabolite interconversion enzymes (46%), transport proteins (7.3%), translation machinery and chaperones (5%), chromatin-binding proteins (<5%), transmembrane signal receptors (<5%), and proteins involved in RNA and DNA metabolism (<5%). According to STRING-db analysis, the deregulated proteins are primarily associated with metabolic processes, which are significantly upregulated in this experimental setting. Notably, these proteins are involved in the metabolism of carboxylic acids, steroids, small molecules, lipids, and fatty acids. The protein–protein interaction (PPI) network revealed at least two dense clusters of interacting proteins. The first cluster mainly comprises xenobiotic-metabolizing enzymes and their associated proteins, predominantly functioning in the cytoplasm (e.g., Cyp2e1, Cyp2b1, Cyp2a1, Cyp2b3, UGT2b1, Ephx2, Slc27a5). The second cluster highlights mitochondrial proteins (e.g., Decr1, Ech1, Hsdl1, Crat) involved in altered mitochondrial activity. A third, less pronounced network includes proteins associated with fatty acid synthesis, isoprenoid biosynthesis, cholesterol metabolism, and partial activation of the β-oxidation pathway (e.g., Fdps, Mvk, Crat, Hmgcs1). One especially interesting group of upregulated proteins is associated with alternative splicing. These include RU1C_RAT (U1 small nuclear ribonucleoprotein C1), B5DFM8_RAT (pre-mRNA splicing factor SPF27), and D4A7J8_RAT (U4/U6 small nuclear ribonucleoprotein Prpf4), among others. All of these are involved in mRNA processing via alternative splicing, suggesting the activation of an energetically efficient and regulatory mechanism for modulating protein function in response to external stimuli.

Finally, several deregulated proteins identified in this analysis are not connected to others via known interactions. While their roles are not discussed in detail here, one exception is made for proteins involved in early apoptotic processes. These proteins align with literature reports on the potential pro-apoptotic effects of opioids on liver cells.

### Proteins Deregulated in the Kidneys

Proteins deregulated in the kidneys are involved in a broader range of biological functions compared to those found in the liver. Using the same selection parameters as applied for the liver data set, the deregulation initially affected up to 20% of all identified proteins. After applying more stringent quantitation criteria (FDR < 0.01, S0 = 0.1), the final group comprised 381 significantly deregulated proteins, accounting for approximately 8% of the proteins selected for quantitative analysis. In contrast to the protein interaction map obtained for liver tissue, the renal data set produced a larger, but more topologically dispersed, PPI network. Multiple interaction modules were present, but each contained a relatively small number of proteins, reflecting the functional diversity of the renal response. Approximately 25% of all deregulated proteins were associated with metabolic and detoxification processes. The remaining proteins were involved in transmembrane and intracellular transport (9.3%), regulation of enzymatic activity (6.9%), cytoskeletal organization (6.6%), transcriptional regulation (<5%), cell adhesion (<5%), and stress responses including heat shock proteins. A distinct, heterogeneous group of deregulated proteins, forming several separate interaction modules, associated with the metabolism of fatty acids, carboxylic acids, amino acids, sulfur compounds, acyl-CoA derivatives, and lipids. We also detected proteins whose activity corresponds to development or inhibition of the apoptotic process, including: ACSL5_RAT (↑), MFGM_RAT(↓), LRP1_RAT(↑), D3ZY71_RAT(↓), CPIN1_RAT (↓), CARF_RAT(↓), RASF2_RAT (↓), NOL3_RAT(↑), PP4C_RAT(↓), MO4L1_RAT(↓) and others (↑ = upregulation, ↓ = downregulation). Pro- and antiapoptotic proteins were not clustered together by STRING-db, as their functions are highly diverse.

Importantly, when the experiment-specific background was applied, no Gene Ontology (GO) Biological Process terms passed the statistical significance threshold for the kidney data set. This result is consistent with the broader, less convergent functional profile of renal DEPs, which did not accumulate in any single dominant biological pathway despite forming multiple smaller interaction modules. A detailed view of the renal interaction network, along with comparison to the hepatic network, is presented in Figure 2.

**Figure 2:**
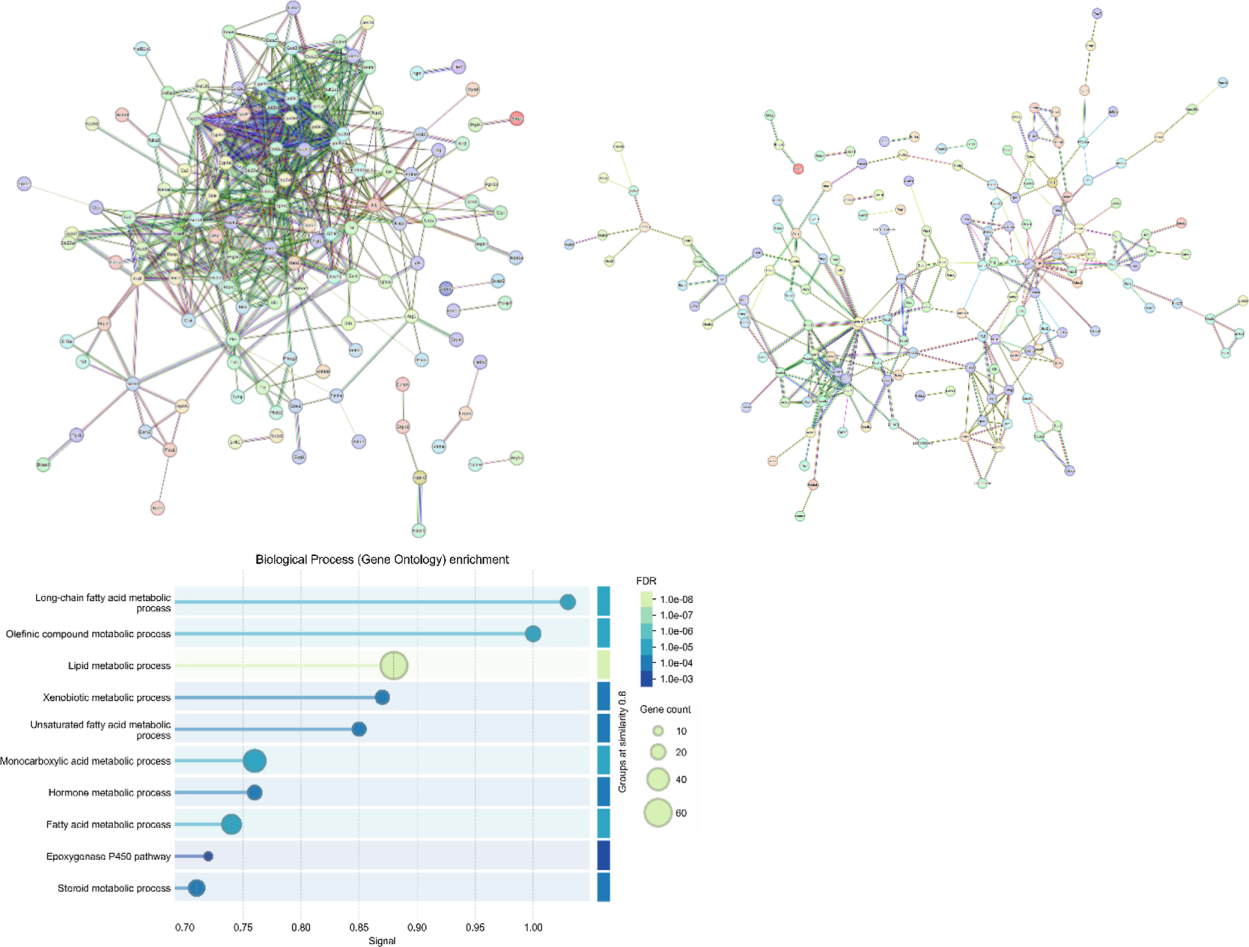
STRING database PPI networks (schematic representation) for deregulated proteins in liver (left upper panel) and kidney tissue (right upper panel), using an experiment-specific background consisting of all proteins identified in this study against the background protein list detected. The bottom panel illustrates the functional roles assigned to interacting proteins by STRING in liver tissue, reflecting a coordinated metabolic response. Details of both PPI networks and GO enrichment can be found in Supporting Information (Figure S11–S13).

### Cross-Tissue Comparison of Morphine-Induced Proteomic Changes

To further assess whether morphine induces coherent or tissue-specific molecular responses, we directly compared the lists of significantly regulated proteins in the liver and the kidney. Using gene-level matching, we identified a subset of proteins that were consistently upregulated in both tissues. This group included classical stress-response and cytoprotective proteins (e.g., Hspa1a, Hspb1, Nqo1), enzymes involved in detoxification and redox balance (Fmo5, Aifm2), and regulators of lipid or metabolic homeostasis (Acot7, Nceh1). GO and Kyoto Encyclopedia of Genes and Genomes (KEGG) enrichment for these commonly upregulated genes highlighted pathways related to response to oxidative stress, protein folding, and xenobiotic metabolism, suggesting that both tissues activate a shared protective program in response to morphine exposure.

**Figure 3:**
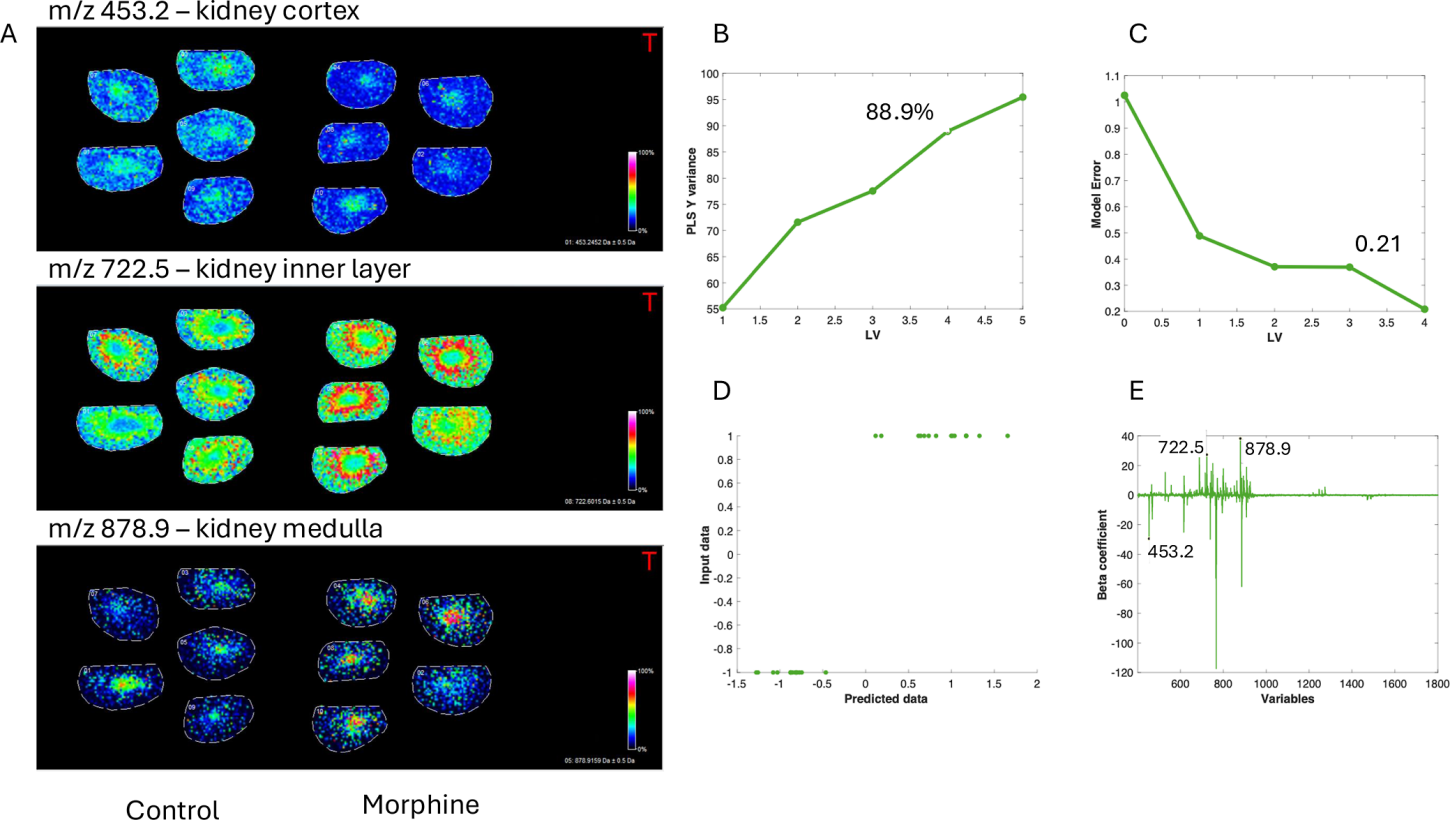
A) Exemplary ions that differentiate control and morphine kidneys. (B) PLS Y variance that is covered by a model with a specific number of LVs. (C) PLS CV error for determination of the correct number of LVs (LV = 4). (D) Predicted (*x*) versus actual (*y*) class assignment of the groups (group separation). (E) PLS Beta Regression Coefficients showing the importance of variables in the discrimination (data for medulla, negative-ion mode).

A smaller set of proteins displayed coherent downregulation in both tissues (e.g., Cyp2c11, Crym, Dhrs7l1). Functional annotations indicated enrichment in metabolic processes, particularly those linked to steroid and lipid metabolism, implying a generalized suppression of selected metabolic pathways under chronic morphine exposure.

In contrast, only a few genes showed opposite regulation between tissues. Four genes (Crat, Cyp4a14, Gstt3, and Prxl2b) were upregulated in the liver yet downregulated in the kidney. These proteins map to pathways controlling fatty acid oxidation, peroxisomal activity, and glutathione-dependent detoxification, suggesting a divergence in metabolic adaptation between the two organs. Notably, no genes were found to be downregulated in the liver while simultaneously upregulated in the kidney. A short summary of the cross-tissue proteome comparison is presented in Table 1.

**Table 1:** Summary of Gene Products Showing Shared or Divergent Regulation Patterns in the Liver and Kidney^a^

interaction type	gene products regulated
liver and kidney ↑	Acot7, Aifm2, Fam91a1, Fmo5, Frrs1, Hspa1a, Hspb1, Kng2, Mettl27, Nceh1, Nqo1
liver and kidney ↓	Crym, Cyp2c11, Dhrs7l1, LOC120102889, Obp3, Thumpd1
liver ↑ kidney ↓	Crat, Cyp4a14, Gstt3, Prxl2b

a Arrows denote the direction of regulation (↑ = UpRegulated; ↓ = DownRegulated). Importantly, we did not detect any gene products that were downregulated in the liver while being upregulated in the kidney

Together, these analyzes demonstrate that while liver and kidney share a substantial portion of the morphine-responsive proteome, each tissue also displays unique regulatory features, particularly in pathways governing lipid utilization and redox homeostasis.

### MSI Results

PLS is a statistical method in the spectrometric field used to model relationships between observed variables, especially useful when the sample size (in this case, biological replicates) is smaller than the number of variables (in our case, the intensities of *m*/*z* peaks corresponding to lipids).^21^ PLS projects the original predictor variables (*m*/*z*) and response variables (peak intensities) into a new latent space and finds latent variables (components) that combine *m*/*z* values to best separate morphine and the control group. To find the best model that separates morphine from the control group, we need to consider the LVs and cross-validation error. In this method, the more new directions (LVs) we include in the model, the better the results will be, as the *Y* variance increases. Nevertheless, we must be careful not to overfit the model, since it will take in more and more “noise.” To find the proper LV value for the model, a CV approach is used. Thus, the minimum CV error indicates the highest LV we can consider without overfitting the model, which may lower its predictive ability. During our study, we sought the optimal parameters to identify *m*/*z* values that discriminate between morphine and the control group in liver and kidney samples. For the kidney, we additionally indicated the kidney cortex, medulla, and inner layer.

For the kidney cortex measured in positive-ion mode, narrower range of *m*/*z* (600–1000) was chosen, as it yielded a model with higher PLS Y variance. The LV = 3 model allowed separation of two classes, reaching a variance of 85.85% and a model error of approximately 0.3. In the morphine kidney cortex, *m*/*z* 820.6, 747.1, 844.8, and 853.8 were upregulated, and *m*/*z* 741.4, 616.2, and 734.8 were downregulated compared to the control cortex. *M/z* 747.4, 734.8, 844.8, and 853.8 were indicated only in two LVs, whereas all LVs indicated the rest of the value. For the kidney cortex measured in negative-ion mode, a wider *m*/*z* range (400–1800) was chosen because it yielded a model with higher PLS Y variance. The LV = 5 model allowed separation of two classes, reaching a variance of 91.65% and a model error of approximately 0.292. In the morphine kidney cortex, 465.4 and 738.6 *m*/*z* were upregulated, while in the control cortex 453.2, 454.2, and 468.2; *m*/*z* were upregulated. Those values were indicated in previous lower LV.

For the kidney medulla measured in positive-ion mode, the LV = 4 model separated two classes, achieving a variance of 87.20% and a model error of approximately 0.35. In the morphine kidney medulla 824 *m*/*z* was upregulated, while in the control medulla, 616.2, 496.3, and 428.0 *m*/*z* were upregulated. Those values were indicated in at least three lower LVs. For the kidney medulla measured in negative-ion mode, LV = 4 model separated two classes, achieving a variance of 88.9% and a model error of approximately 0.194686. In the morphine kidney medulla, 722.5 and 878.9 *m*/*z* were upregulated, while in the control cortex 453.2, 615.1, *m*/*z* were upregulated. Those values were also indicated at different LVs. Examples of the results are shown in Fig. 3.

For the kidney inner layer measured in positive-ion mode, the LV = 4 model separated two classes, achieving a variance of 89.11% and a model error of approximately 0.2017. In the control cortex, 616.2 and 664.1 *m*/*z* were upregulated. Those values were indicated in at least three lower LVs. In the kidney inner layer negative-ion mode, LV = 3 model allowed separation of two classes, achieving a variance of 90.2% and a model error of approximately 0.221. In the morphine kidney inner layer, 885.5 and 750.5 *m*/*z* were upregulated, while in the control cortex, 453.2 and 615.1 *m*/*z* were upregulated. Those values were indicated in at least two different LVs. Indicated peaks were identified based on MS/MS spectra, or literature data.

The interesting problem in this context is that, for the liver analysis, we were unable to create a model that separated the morphine and control groups. It should be noted that, using MALDI–MSI approach, we can observe only lipids that ionize under these special conditions with this ion source and chosen matrix, hence important molecules may remain undiscovered in this context.

## Discussion

The present study provides complementary proteomic and lipidomic evidence describing the early molecular response of liver and kidney tissues to morphine exposure at a therapeutic doses. Both organs exhibited clear proteomic alterations, yet their lipidomic profiles differed markedly: no detectable lipid changes were observed in the liver, whereas the kidney displayed early lipidomic shifts that likely reflect downstream consequences of its proteomic deregulation.

The liver is generally considered resilient to short-term opioid exposure in healthy individuals. However, in patients with impaired liver function, opioid therapy requires careful monitoring, dose adjustment, and attention to drug–drug interactions.^1,4^ The limited number of fundamental studies exploring early hepatic responses to morphine underscores the relevance of our findings. Consistent with earlier work in rodent models using similar dosing regimens,^8^ morphine triggered proteomic changes related to oxidative stress and apoptosis, although previous studies did not use proteomic techniques and focused on glutathione depletion, viability assays and serum enzyme markers.^22^ Our data set supports those observations by revealing deregulation of both pro- and antiapoptotic proteins, suggesting dynamic equilibrium between injury and early compensatory mechanisms. A longer exposure period may be required to determine whether apoptosis progresses or is successfully counteracted.

One of the most intriguing findings in the liver was the upregulation of proteins involved in alternative splicing (e.g., RU1C_RAT, B5DFM8_RAT, D4A7J8_RAT, Q4KLI4_RAT). Although untargeted proteomics cannot directly identify specific isoforms, the upregulation of spliceosomal components suggests an adaptive regulatory response. Similar splicing-related changes have been observed in other contexts, such as anticancer therapies, but typically emerge after longer exposures.^23,24^ Our observations may therefore represent an early stage of this regulatory activity.

Despite deregulation of proteins involved in lipid metabolism, the hepatic lipidome remained stable. This aligns with literature indicating transient changes in hepatic triglycerides shortly after morphine or Stadol administration, which normalize rapidly within 36 h.^25^ Our MSI results after 10 days of exposure corroborate these reports.

Kidneys, cooperating with the liver in xenobiotic processing, showed a markedly different pattern. Previous studies of opioid-induced renal injury have focused mainly on severe, late-stage damage, detected through histopathology, and selected biochemical markers.^26^ To our knowledge, the present work provides the first proteomic characterization of early renal molecular events following morphine exposure.

Initial analysis revealed widespread proteomic disruption, prompting us to adopt more stringent statistical thresholds, resulting in 381 significantly deregulated proteins. Unlike the liver, the renal PPI network consisted of multiple small modules rather than coherent clusters, indicating a broad, multidirectional, but nontargeted response. Approximately 25% of deregulated proteins participated in metabolism and detoxification, supporting the kidneys’ auxiliary role in biotransformation.^3^ Numerous proteins linked to apoptotic pathways were identified, yet both pro- and antiapoptotic regulators were up- or downregulated, suggesting simultaneous activation of injury- and repair-related mechanisms. Further studies, particularly those using multiple time points, may provide deeper insight into this inconsistent observation.

Signs of oxidative stress were evident, including deregulation of proteins such as CSRP2_RAT, NIBA1_RAT, CY24A_RAT, glutathione transferases, NQO1_RAT, LEG3_RAT, and PTMA_RAT, consistent with previous reports.^26^ These changes coincided with alterations in cytoskeletal components (e.g., CAPG_RAT, AIF1_RAT, BI2L1_RAT, WASH1_RAT), potentially showing initial steps toward structural and functional impairment observed in later renal pathologies, including podocytopathies^27^ or fibroblast dysfunction.^28^

The cross-tissue overlap of morphine-regulated proteins provides additional insight into the systemic versus organ-specific nature of the drug’s action. The coordinated upregulation of stress-response chaperones (Hspa1a, Hspb1) and redox-regulating enzymes (Nqo1, Aifm2) in both liver and kidney suggests that chronic morphine exposure triggers a conserved protective program aimed at counteracting oxidative stress. These findings align well with previous reports describing increased production of reactive oxygen species (ROS) and impaired antioxidant capacity during prolonged opioid administration.

Conversely, the group of proteins consistently downregulated in both tissues (e.g., Cyp2c11, Crym) points toward a generalized suppression of metabolic capacity. Enrichment analyzes implicate pathways involved in lipid and steroid processing. This systemic metabolic downshifting may reflect an adaptive response to reduced energy availability or altered hormonal signaling under repeated morphine treatment.

Of particular interest is the presence of a small set of proteins that are oppositely regulated in the liver and the kidney. Enzymes such as Cyp4a14, Crat, and Gstt3 participate in fatty acid oxidation and detoxification pathways. Their upregulation in the liver but downregulation in the kidney may indicate that the liver compensates for increased metabolic load. In contrast, the kidney, which may be more susceptible to ROS, attenuates these pathways to limit further stress. Such divergence underscores the importance of evaluating multiple tissues when characterizing pharmacological or toxicological effects of morphine. Overall, integrating GO/KEGG-based functional annotation with statistical proteomics supports a model in which chronic morphine induces (i) a shared adaptive stress-response program across organs and (ii) tissue-specific metabolic remodeling, particularly pronounced in pathways associated with lipid metabolism and antioxidant defenses.

To complement the proteomic findings, lipidomic imaging of kidney sections revealed deregulation of selected phosphatidylcholines (PC 36:4, PC 36:2, PC 38) and LysoPC, consistent with altered membrane remodeling and previously noted associations between PC metabolism and renal injury.^29,30^ The elevation of PE plasmalogens, which mitigate oxidative damage,^11^ may reflect a protective response. Lower heme *B* signal intensity (616.2/615.1 *m*/*z*) further suggests localized tissue disruption, in agreement with previous MSI observations.^31^

Taken together, our findings indicate that both organs undergo early proteomic adaptations to morphine exposure; however, the liver demonstrates markedly greater resilience and more targeted changes toward xenobiotic processing. Hepatic proteomic shifts remain functionally coordinated and do not translate into lipidomic alterations. In contrast, the kidney exhibits broad, multifunctional deregulation accompanied by detectable lipidomic changes, suggesting earlier progression toward tissue stress and damage.

In conclusion, the perception that opioids exert minimal toxic effects on liver and kidney tissues may be overly optimistic. While healthy organs may compensate for short-term exposure, our proteomic and lipidomic data reveal early molecular disturbances in both tissues. In individuals with pre-existing hepatic or renal conditions, or those undergoing multidrug therapy, careful monitoring remains essential. With further validation, proteomic markers may support personalized assessment of organ status during opioid treatment and help mitigate the risk of progressive organ injury.

### Future Perspectives

As the present study provides a preliminary yet coherent molecular overview, several directions for future research can be proposed. First, extending the experimental design to include multiple time points and a range of morphine doses within the therapeutically relevant interval would greatly enhance understanding of the temporal dynamics and dose-dependence of organ responses. Such an approach would help identify key biomarkers reflecting early oxidative stress, apoptosis, or metabolic impairment, and determine how these signals evolve as a function of exposure duration, dose, and tissue type.

Second, independent validation of the most significantly deregulated proteins and lipids, using techniques such as Western blotting, ELISA, or targeted MS, will be essential to establish their robustness and potential utility as biomarkers. These efforts may enable the development of proteomic panels suitable for individualized monitoring of hepatic and renal function during opioid therapy.

Further mechanistic studies, although beyond the scope of the present work, would be valuable for elucidating upstream signaling events and dynamic interactions within the identified pathways, including receptor-level regulation, enzyme activities, and intercellular communication. Approaches such as NMR-based metabolomics or functional imaging, combined with histopathological analysis, could help link molecular findings to early manifestations of tissue injury.

Together, these future investigations may deepen our understanding of morphine-induced organ stress, support translational biomarker development, and contribute to safer, personalized strategies for opioid administration.

## Supplementary Material





## Data Availability

The mass spectrometry proteomics data have been deposited to the ProteomeXchange Consortium via the PRIDE partner repository with the data set identifiers: for DDA experiments PXD067999 and 10.6019/PXD067999, for DIA experiments PXD068084.

## Notes

Table presenting both: down- and up-regulated proteins (containing: –log_2_(*p*-values), differences, protein groups, protein names, genes, and protein description) in both tissues: Liver and kidney (XLSX)
Figure S1: Kernel density plots of log_2_-transformed protein intensities for all liver samples. Figure S2: Venn diagram of proteins detected in liver tissue before filtering steps. Figure S3*:* Box-and-whisker plots of log_2_-transformed protein intensities for all liver samples. Figure S4: Pearson correlation matrix of log_2_-transformed protein intensities across all liver samples. Figure S5: Principal component analysis (PCA) of liver samples based on log_2_-transformed protein intensities. Figure S6: Kernel density plots of log_2_-transformed protein intensities for all kidney samples. Figure S7: Venn diagram of proteins detected in kidney tissue before filtering steps. Figure S8*:* Box-and-whisker plots of log_2_-transformed protein intensities for all kidney samples. Figure S9: Pearson correlation matrix of log_2_-transformed protein intensities across all kidney samples. Figure S10: Principal component analysis (PCA) of kidney samples based on log_2_-transformed protein intensities. Figure S11: STRING database PPI networks for deregulated proteins in liver. Figure S12: STRING database PPI networks for deregulated proteins in kidney. Figure S13: Biological process (GO) enrichment found for liver-derived regulated proteins (PDF)
