# Supplementary material for: Liver and Kidney Tissues Under Opioid Exposure: Rewriting and Old Story Through Proteomics and MALDI–MSI
Source: J Proteome Res. 2026 Jan 24;25(2):995–1004. doi: 10.1021/acs.jproteome.5c00764 (PMC13296724; doi:10.1021/acs.jproteome.5c00764)
Supplement: Supplementary file 2 [file pr5c00764_si_002.pdf]

Supplementary materials to:

Liver and Kidney Tissues Under Opioid Exposure: Rewriting and Old Story Through Proteomics and MALDI-MSI.

Malgorzata Hopcias,[1] Paulina Kret,[1] Jolanta H. Kotlinska,[2] Pawel Link-Lenczowski,[3,4] Anna Bodzon-Kulakowska,[1] Piotr Suder[1,]*

[1]University of Krakow, Department of Analytical Chemistry and Biochemistry, Mickiewicza 30 ave., 30-059 Krakow, Poland

[2]Medical University of Lublin, Department of Pharmacology and Pharmacodynamics, Chodzki 4A st. 20-093 Lublin, Poland.

[3]Department of Medical Physiology, Faculty of Health Sciences, Jagiellonian University Medical College, 31-126 Krakow, Poland

[4]Center for the Development of Therapies for Civilization and Age-Related Diseases, Jagiellonian University Medical College, 31-066 Krakow, Poland

*corresponding author (e-mail: psuder@agh.edu.pl)

Supporting information table of contents (TOC):

1. S1_table1.xlsx – table presenting both: down- and up-regulated proteins (containing: -log_2(p-values), differences, protein groups, protein names, genes and protein description) in both tissues: liver and kidney

2. Supplementary_QC doc file containing:

Figure S1: Kernel density plots of log_2-transformed protein intensities for all liver samples

Figure S2: Venn diagram of proteins detected in liver tissue before filtering steps

Figure S3: Box-and-whisker plots of log_2-transformed protein intensities for all liver samples

Figure S4: Pearson correlation matrix of log_2-transformed protein intensities across all liver samples.

Figure S5: Principal component analysis (PCA) of liver samples based on log_2-transformed protein intensities.

Figure S6: Kernel density plots of log_2-transformed protein intensities for all kidney samples

Figure S7: Venn diagram of proteins detected in kidney tissue before filtering steps

Figure S8: Box-and-whisker plots of log_2-transformed protein intensities for all kidney samples

Figure S9: Pearson correlation matrix of log_2-transformed protein intensities across all kidney samples.

Figure S10: Principal component analysis (PCA) of kidney samples based on log_2-transformed protein intensities.

Figure S11: STRING database PPI networks for deregulated proteins in liver

Figure S12: STRING database PPI networks for deregulated proteins in kidney

Figure S13: Biological process (GO) enrichment found for liver-derived regulated proteins

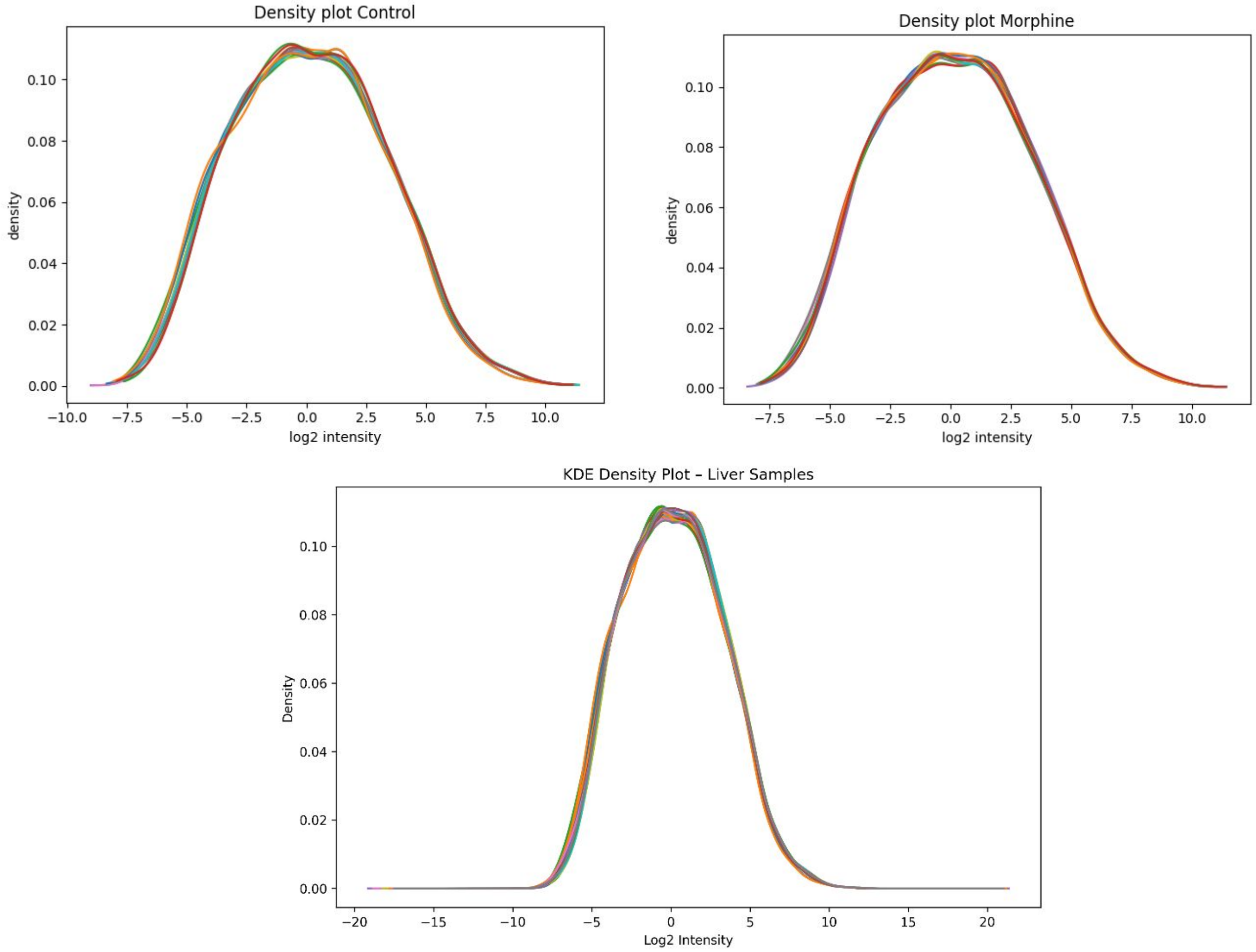

Figure S1: Kernel density plots of log_2-transformed protein intensities for all liver samples (upper left: control group, upper right: morphine-treated group, bottom: combined dataset). The distributions for both groups show nearly identical shapes and central tendencies, indicating stable global signal levels across runs and effective median-based normalization prior to differential expression analysis.

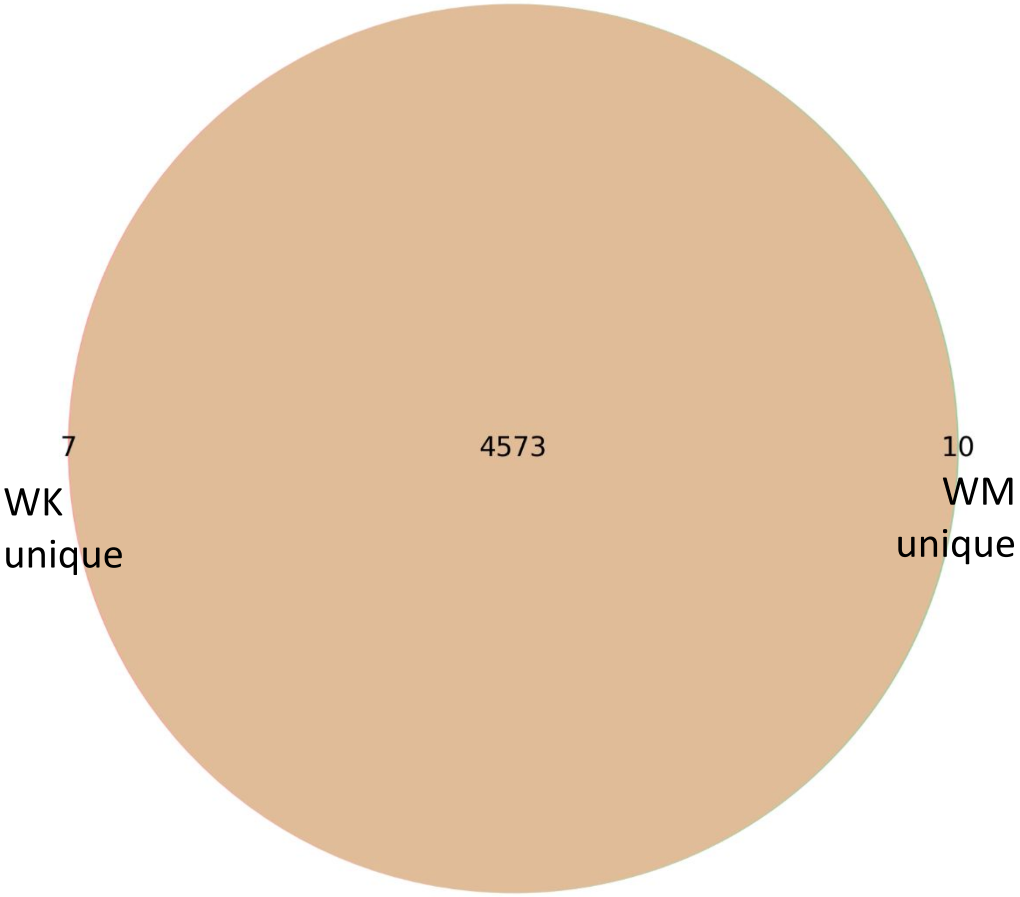

Figure S2. Venn diagram of proteins detected in liver tissue before filtering steps (Perseus, matrix 1). A total of 4573 proteins were identified in both control and morphine-treated groups, whereas only 7 and 10 proteins were uniquely observed in WK (liver control) and WM (liver morphine) groups, respectively. This confirms highly consistent identification depth across samples and demonstrates stable LC–MS performance.

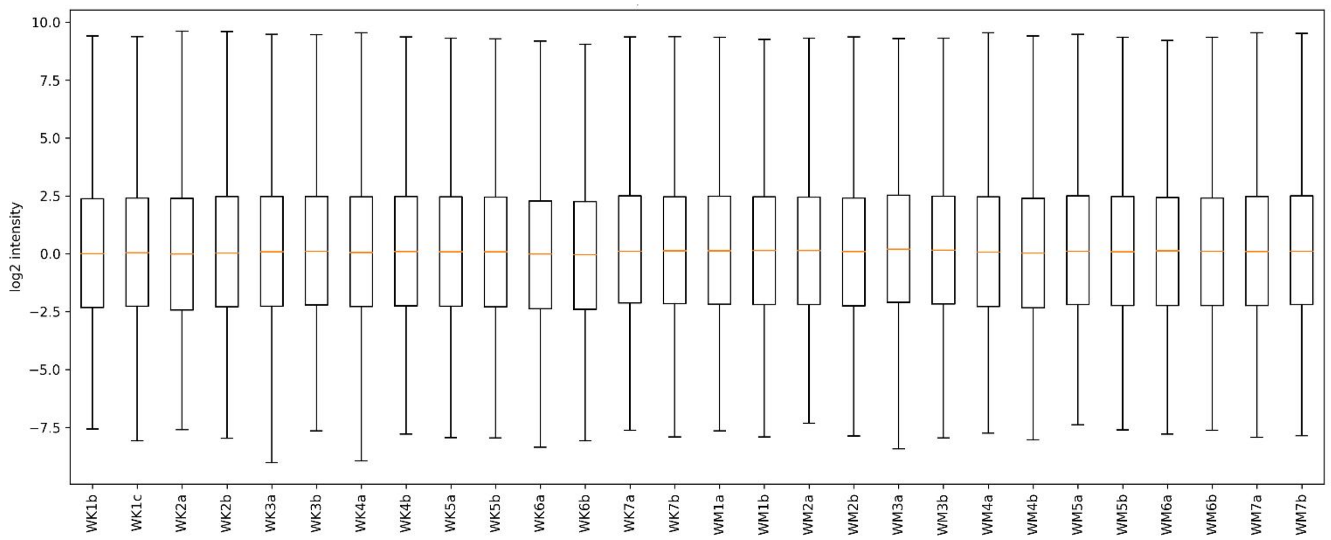

Figure S3. Box-and-whisker plots of log_2-transformed protein intensities for all liver samples (WK = liver controls, WM = liver morphine). Each box displays the interquartile range (IQR), with whiskers extending to 1.5×IQR; outliers were removed for clarity. Median intensity levels are highly comparable between samples, and the overall distribution shapes are uniform across all LC–MS injections. This consistency demonstrates stable quantitative performance of the DIA workflow and confirms the absence of technical drifts or injection-related biases.

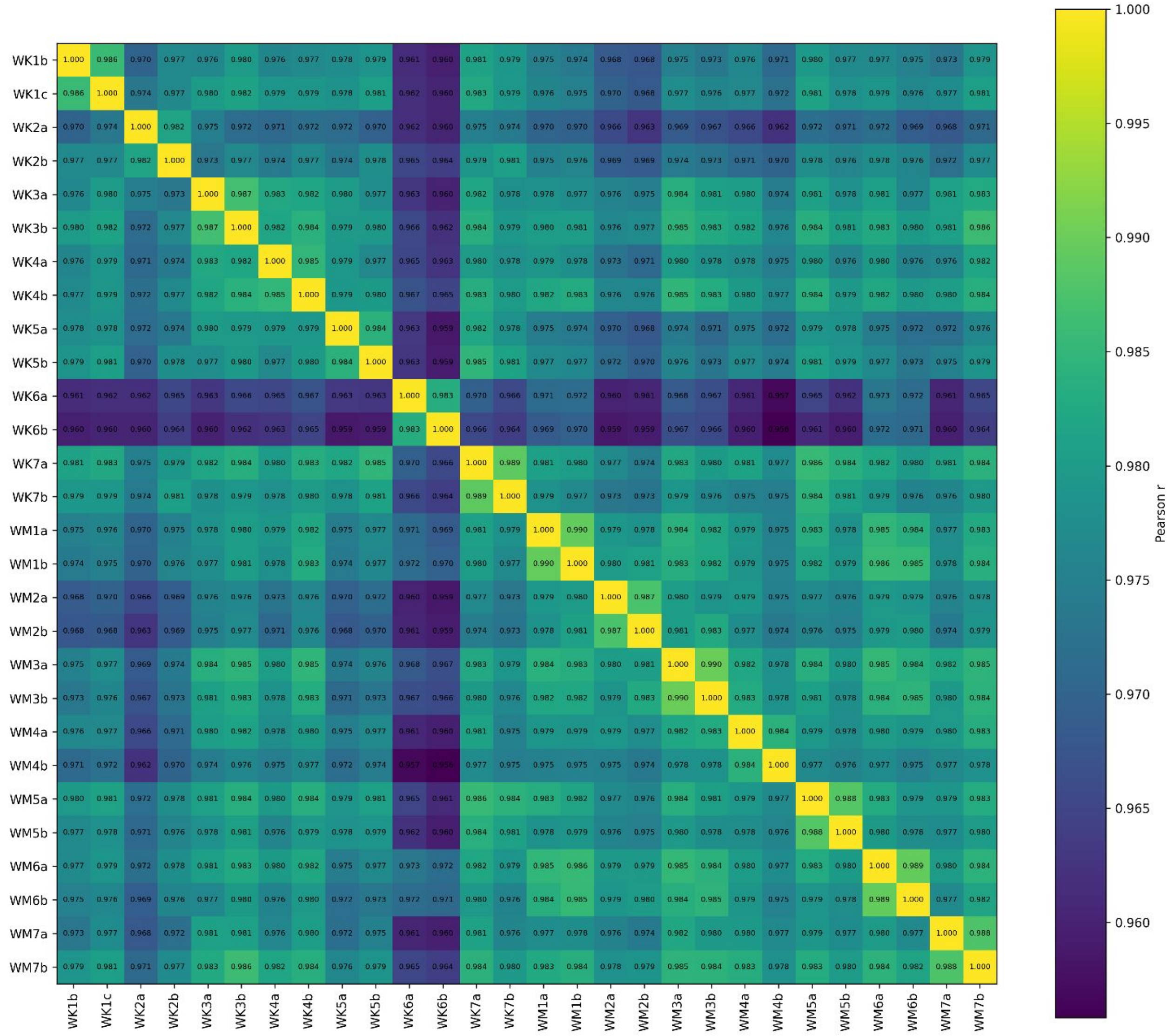

Figure S4: Pearson correlation matrix of log_2-transformed protein intensities across all liver samples (WK = liver control, WM = liver morphine). High correlation coefficients, particularly between technical replicates, confirm high LC–MS stability and reproducibility. The color scale was adjusted to maximize the dynamic range of the color gradient, with the Pearson correlation coefficient range set between 0.95 and 1.00. They are also given in each box for corresponding samples.

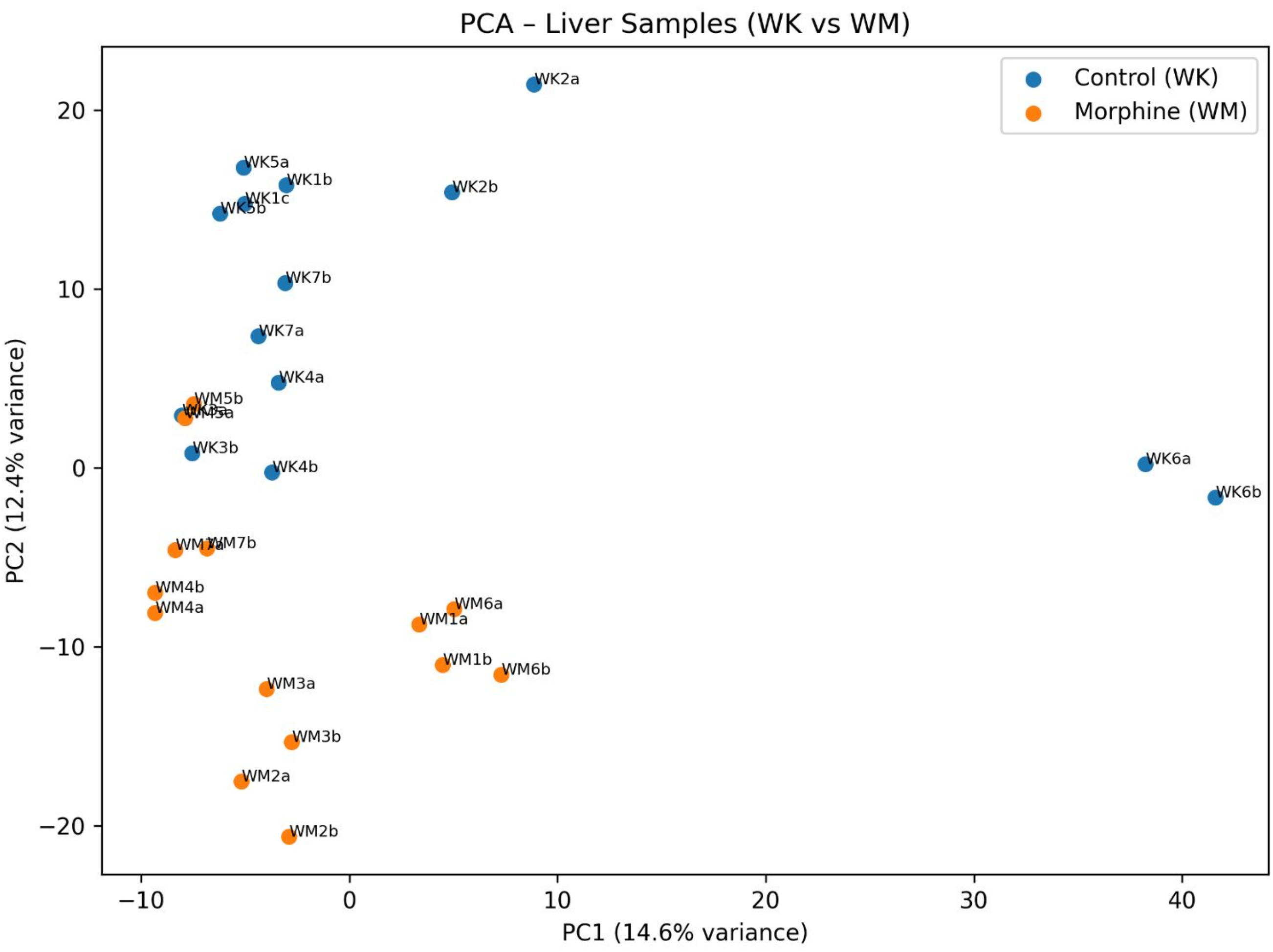

Figure S5: Principal component analysis (PCA) of liver samples based on log_2-transformed protein intensities. The first two principal components shown 14.6% (PC1) and 12.4% (PC2) of the total variance and together revealed a clear, though partial, separation between control and morphine-treated animals, indicating biologically meaningful treatment-related effects on the liver proteome. Technical replicates clustered tightly, demonstrating high analytical stability of the LC–MS workflow and reproducibility of sample preparation. No technical outliers or aberrant clustering patterns were detected, confirming the overall robustness and homogeneity of the dataset.

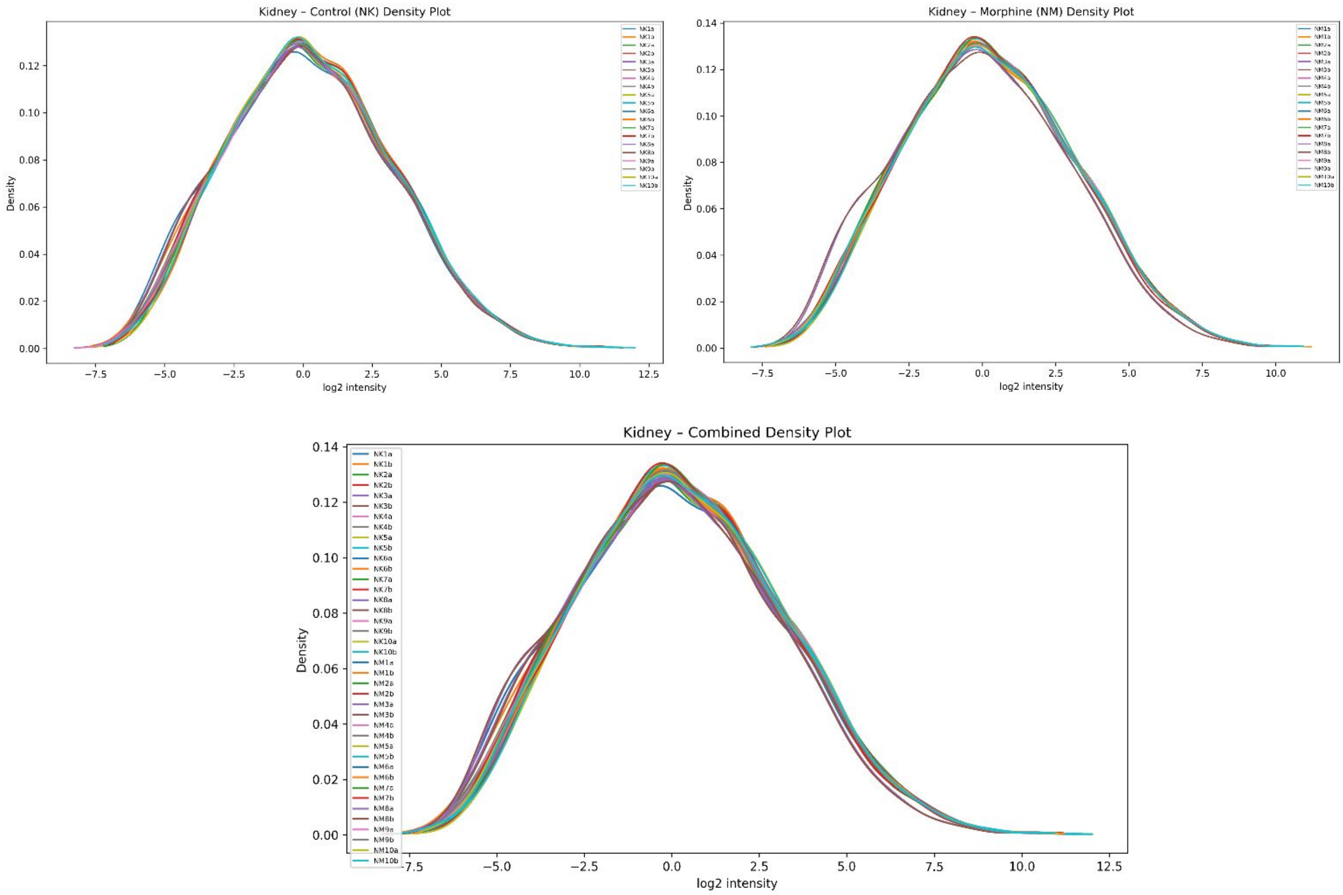

Figure S6: Kernel density plots of log_2-transformed protein intensities for all kidney samples (upper left: control group; upper right: morphine-treated group; bottom: combined dataset). Compared to liver tissue, kidney samples exhibited a slightly narrower and more peaked (“cone-shaped”) intensity distribution, consistent with the lower cellular and functional heterogeneity of renal tissue. Importantly, all kidney samples displayed nearly identical distribution profiles, confirming excellent consistency and stability of the LC–MS workflow.

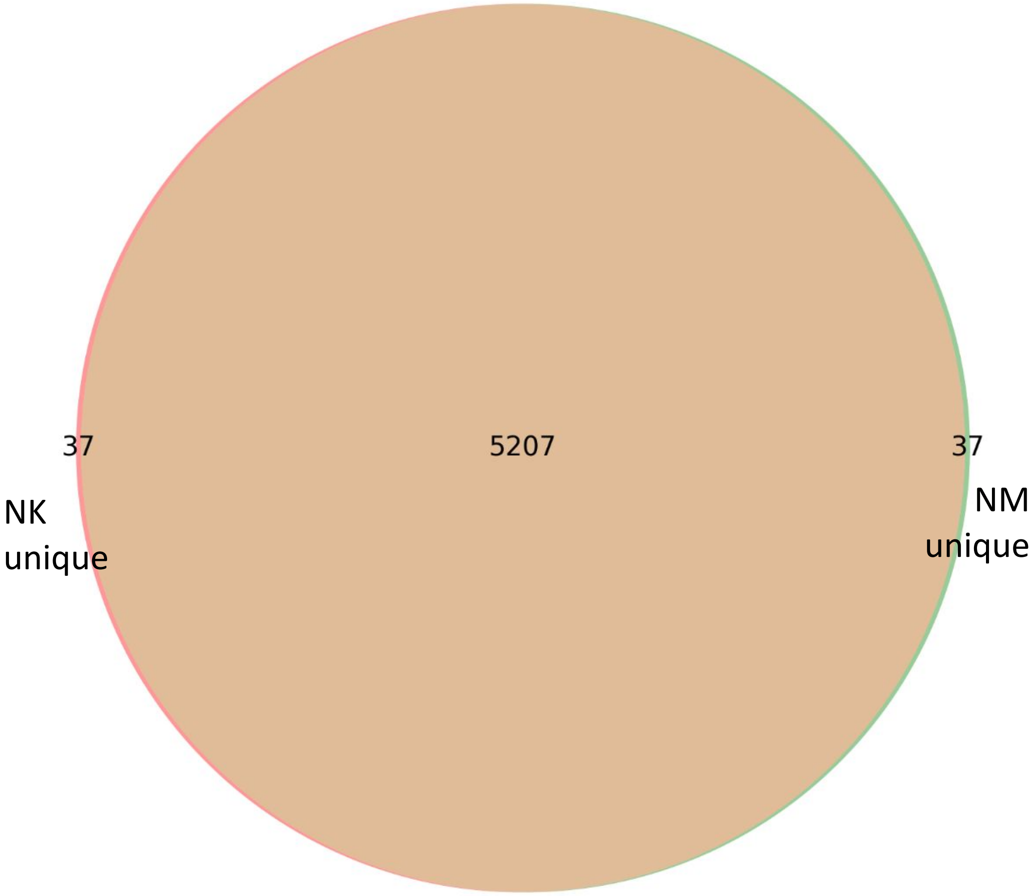

Figure S7: Venn diagram of proteins detected in kidney samples prior to any filtering (Matrix 1). A total of 5207 proteins were identified in both control and morphine-treated groups, whereas only 37 proteins were uniquely present in NK (kidney control) samples with the same quantity of unique proteins in NM (kidney morphine) samples. This overlap demonstrates excellent consistency of protein identifications and confirms the robustness of the LC–MS workflow.

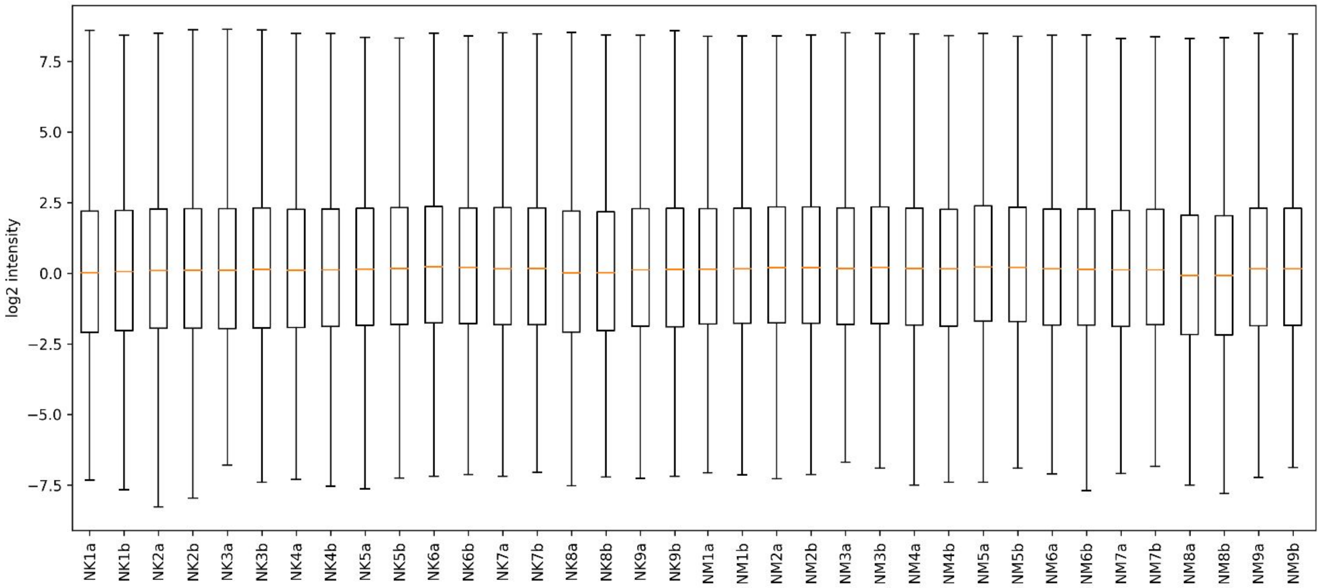

Figure S8: Box-and-whisker plots of log_2-transformed protein intensities for all kidney samples (NK = kidney controls, NM = kidney morphine). Each box displays the interquartile range (IQR), with whiskers extending to 1.5×IQR; outliers were removed for clarity. Median intensity levels are highly comparable between samples, and the overall distribution shapes are uniform across all LC–MS injections. This consistency demonstrates stable quantitative performance of the DIA workflow and confirms the absence of technical drifts or injection-related biases.

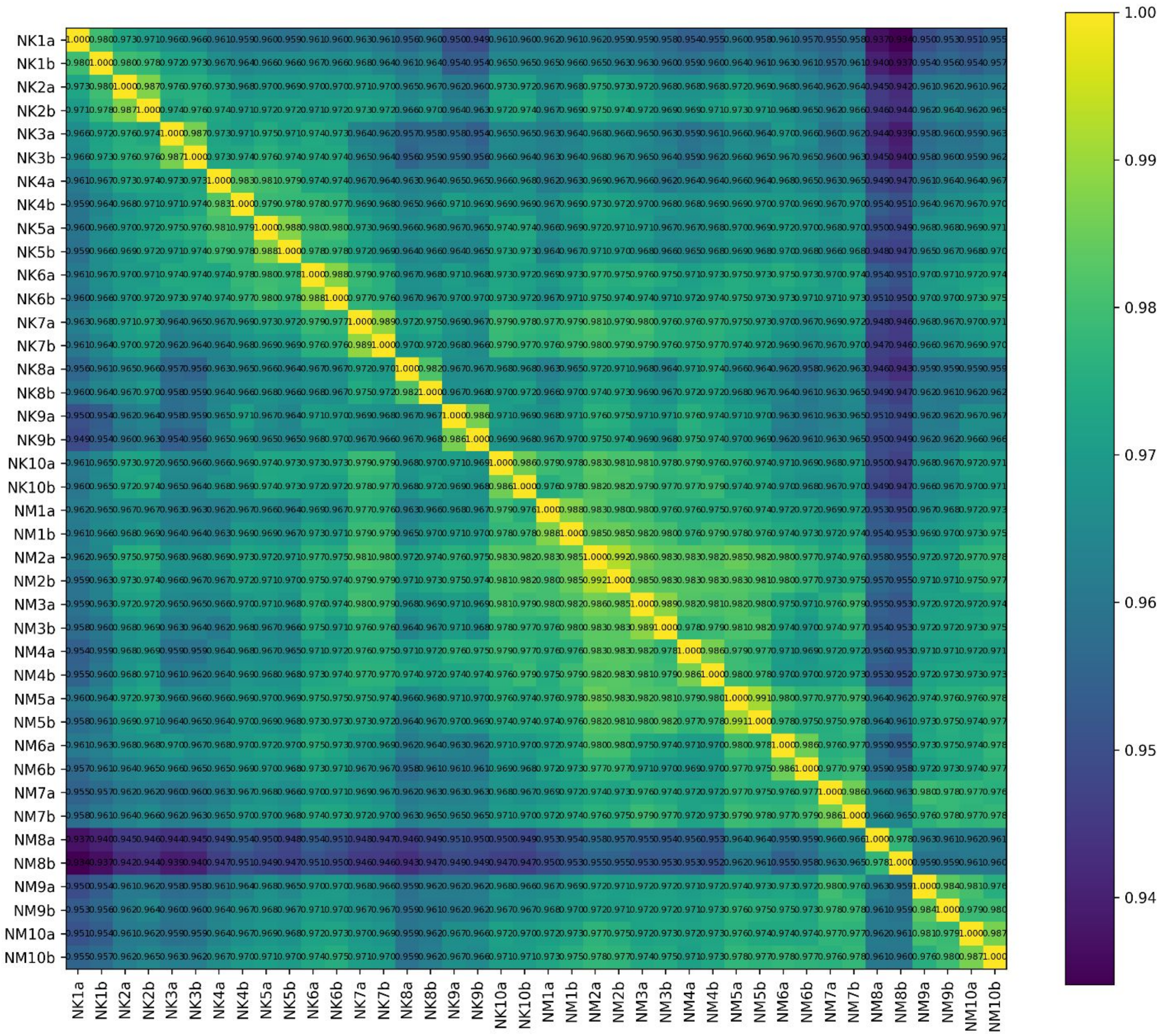

Figure S9: Pearson correlation matrix of log_2-transformed protein intensities across all kidney samples (NK = kidney control, NM = kidney morphine). High correlation coefficients, particularly between technical replicates, confirm high LC–MS stability and reproducibility. The color scale was adjusted to maximize the dynamic range of the gradient, with the Pearson correlation coefficient range set between 0.93 and 1.00. They are also given in each box for corresponding samples.

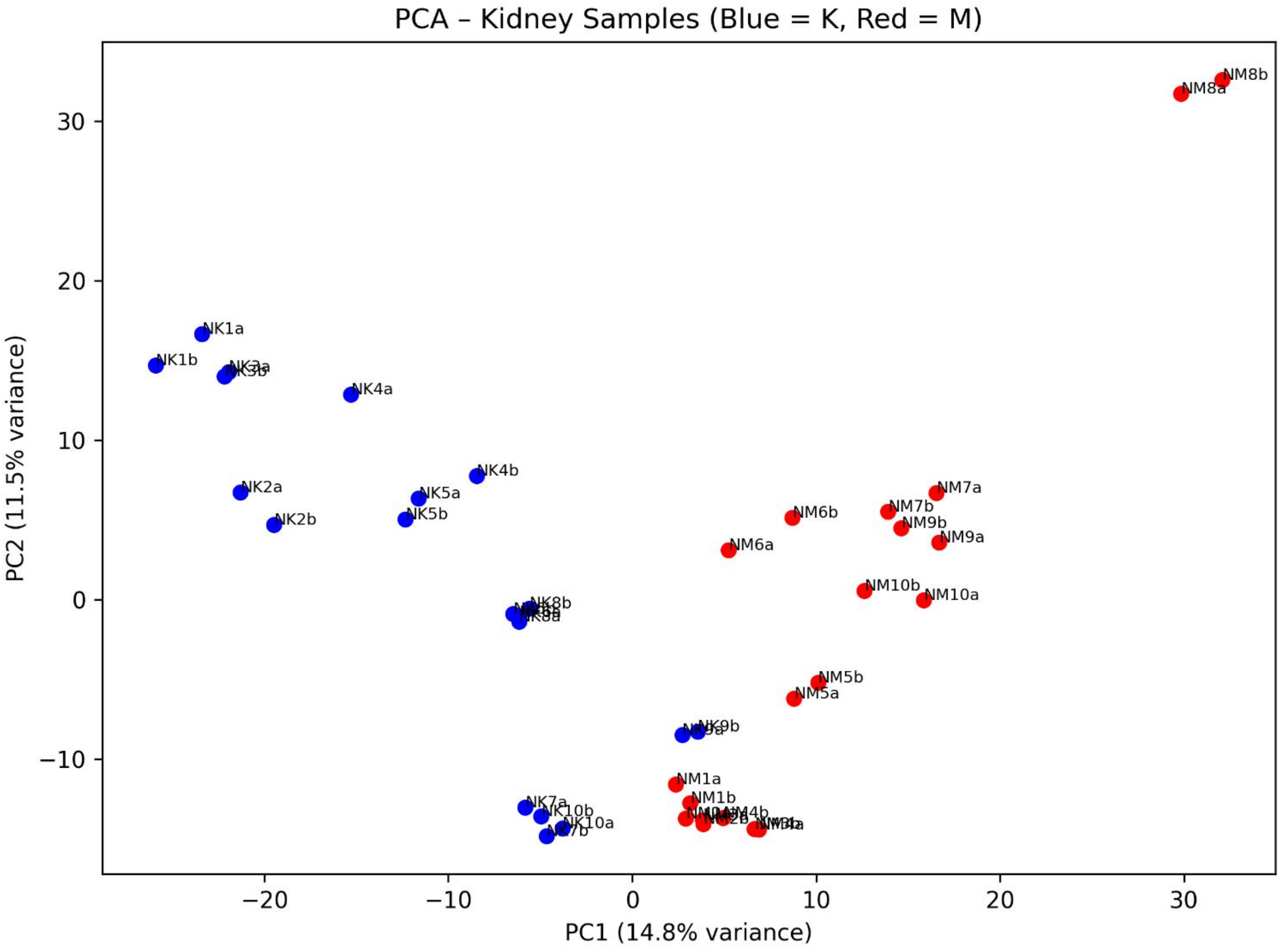

Figure S10: Principal component analysis (PCA) of kidney samples based on log_2-transformed protein intensities. The first two principal components explained 14.8% (PC1) and 11.5% (PC2) of the total variance. Sample clustering revealed a clear left–right separation, with control samples (NK) located predominantly on the left side of the plot and morphine-treated samples (NM) on the right, indicating distinct treatment-related alterations in the renal proteome. Technical replicates clustered tightly, demonstrating high reproducibility of the LC–MS workflow and consistency of sample preparation. No technical outliers or irregular clustering patterns were observed, confirming the robustness and homogeneity of the dataset.

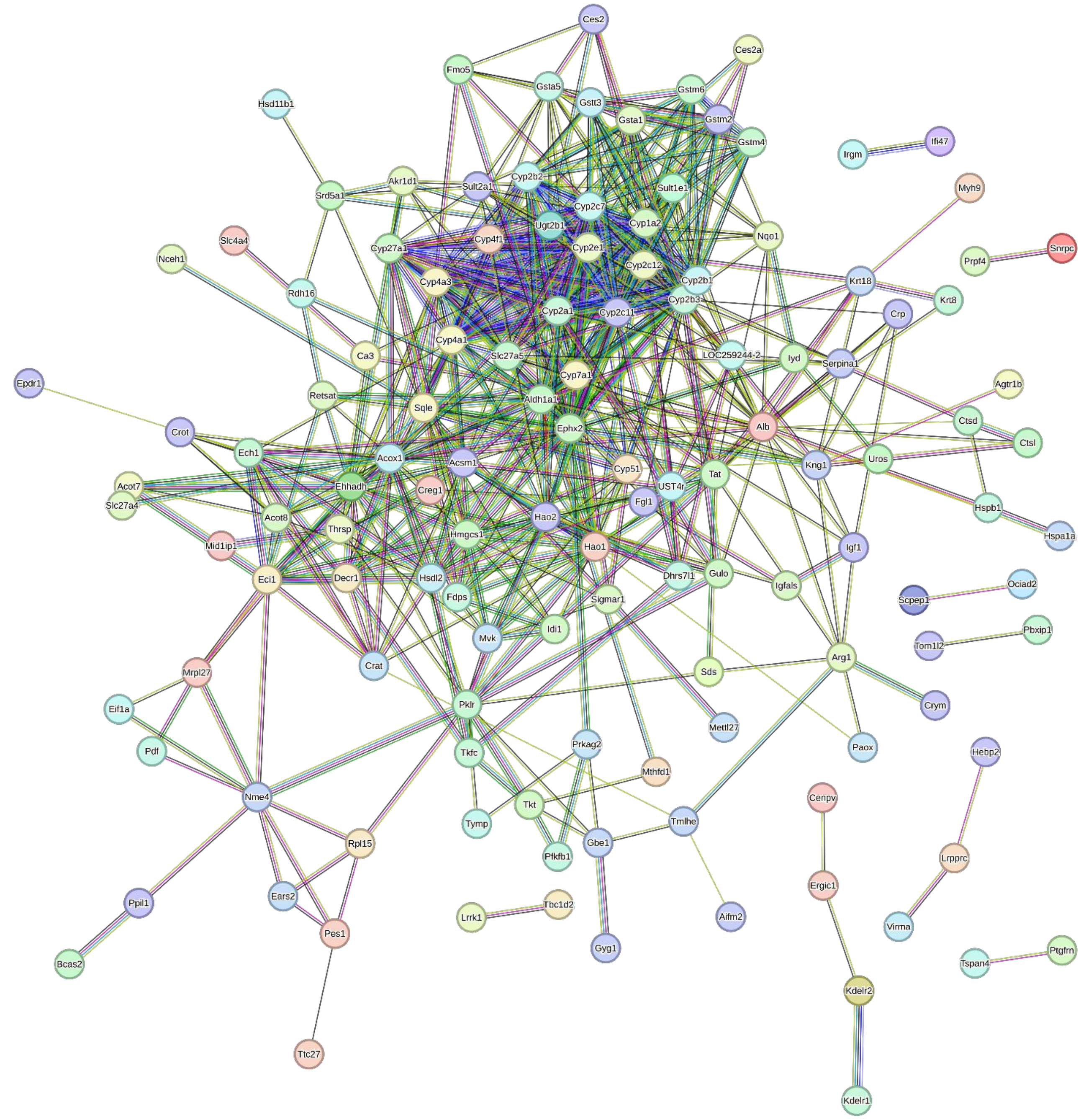

Figure S11: STRING database PPI networks for deregulated proteins in liver

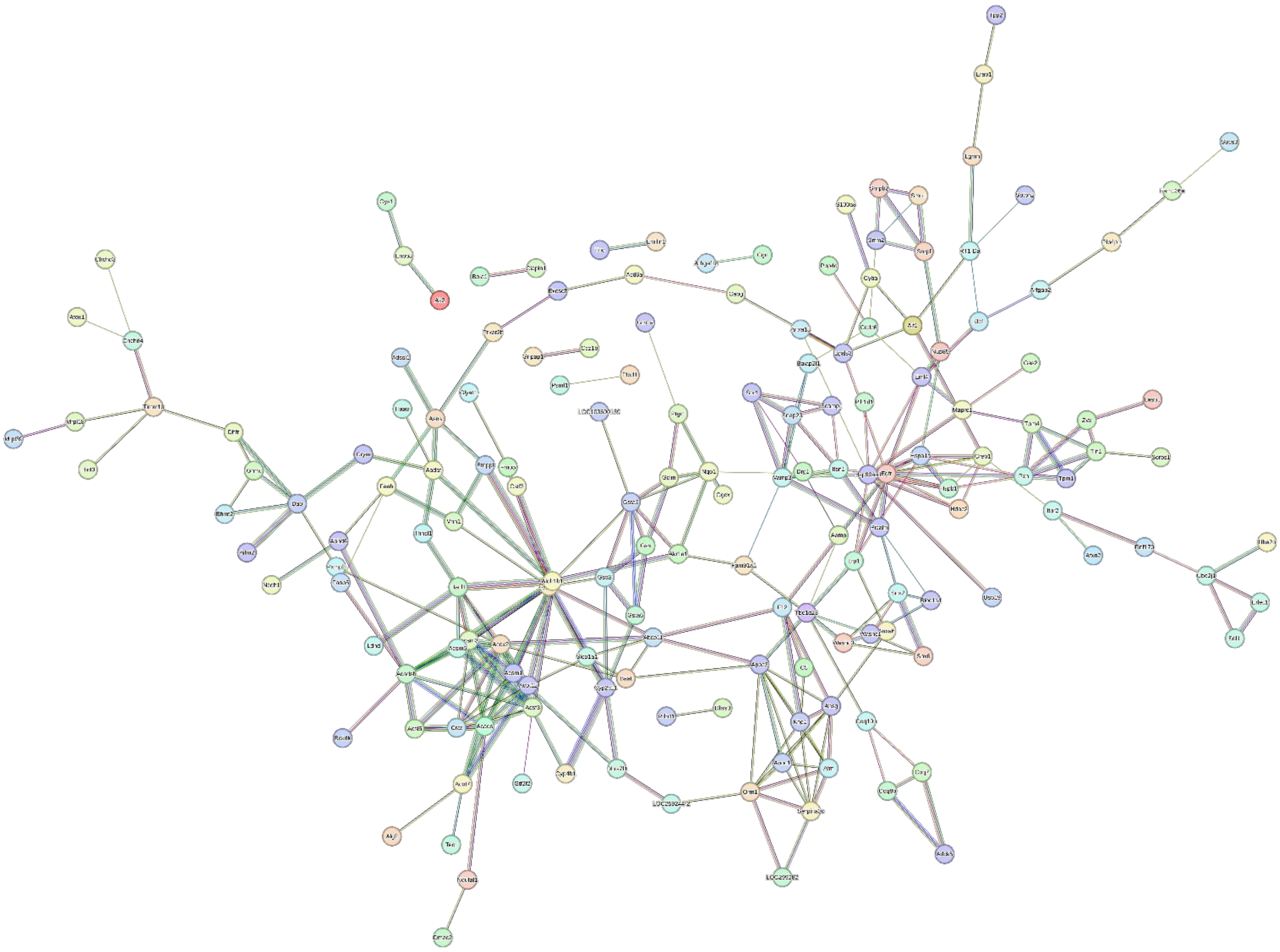

Figure S12: STRING database PPI networks for deregulated proteins in kidney

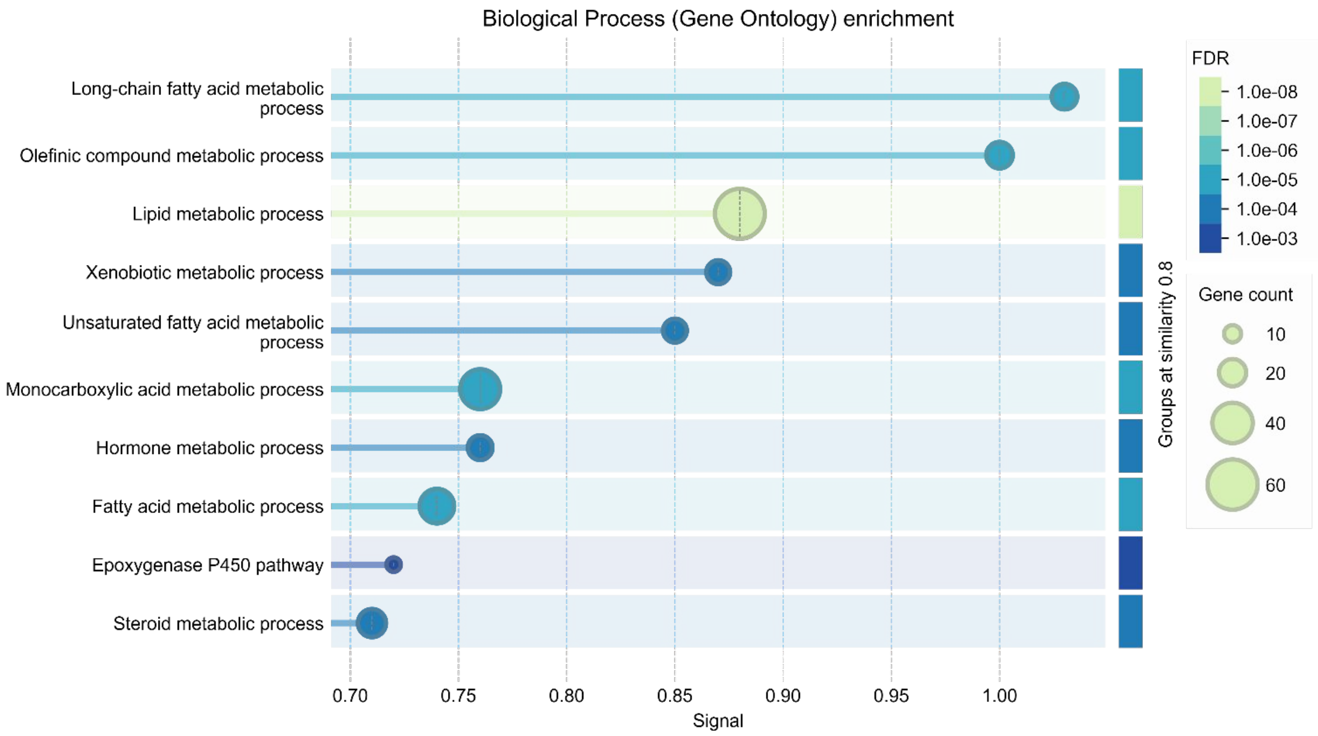

Figure S13: Biological process (GO) enrichment found for liver-derived regulated proteins
